# The tumour suppressor SOX11 is associated with improved survival among high grade epithelial ovarian cancers and is regulated by reversible promoter methylation

**DOI:** 10.1186/1471-2407-11-405

**Published:** 2011-09-24

**Authors:** Sandra Sernbo, Elin Gustavsson, Donal J Brennan, William M Gallagher, Elton Rexhepaj, Frida Rydnert, Karin Jirström, Carl AK Borrebaeck, Sara Ek

**Affiliations:** 1 Department of Immunotechnology, Lund University, Lund, Sweden; 2 CREATE Health, Lund University, BMC D13, 221 84 Lund, Sweden; 3 UCD School of Biomolecular and Biomedical Science, UCD Conway Institute, University College, Dublin, Ireland; 4 Department of Clinical Sciences, Pathology, Lund University Hospital, Lund University, Lund, Sweden

**Keywords:** SOX11, EOC, DNA methylation, epigenetic regulation

## Abstract

**Background:**

The neural transcription factor SOX11 has been described as a prognostic marker in epithelial ovarian cancers (EOC), however its role in individual histological subtypes and tumour grade requires further clarification. Furthermore, methylation-dependent silencing of SOX11 has been reported for B cell lymphomas and indicates that epigenetic drugs may be used to re-express this tumour suppressor, but information on SOX11 promoter methylation in EOC is still lacking.

**Methods:**

SOX11 expression and clinicopathological data was compared using χ^2 ^test in a cohort of 154 cases of primary invasive EOC. Kaplan-Meier analysis and the log rank test were applied to evaluate ovarian cancer-specific survival (OCSS) and overall survival (OS) in strata, according to SOX11 expression. Also, the methylation status of the SOX11 promoter was determined by sodium bisulfite sequencing and methylation specific PCR (MSP). Furthermore, the effect of ectopic overexpression of SOX11 on proliferation was studied through [3H]-thymidine incorporation.

**Results:**

SOX11 expression was associated with an improved survival of patients with high grade EOC, although not independent of stage. Further analyses of EOC cell lines showed that SOX11 mRNA and protein were expressed in two of five cell lines, correlating with promoter methylation status. Demethylation was successfully performed using 5'-Aza-2'deoxycytidine (5-Aza-dC) resulting in SOX11 mRNA and protein expression in a previously negative EOC cell line. Furthermore, overexpression of SOX11 in EOC cell lines confirmed the growth regulatory role of SOX11.

**Conclusions:**

SOX11 is a functionally associated protein in EOC with prognostic value for high-grade tumours. Re-expression of SOX11 in EOC indicates a potential use of epigenetic drugs to affect cellular growth in SOX11-negative tumours.

## Background

EOC is a heterogeneous disease compromising many histological subtypes including clear cell, mucinous, endometrioid and serous carcinoma, which are sub-classified into high- and low-grade [1]. Differences in survival between the histological subtypes have been observed, with mucinous and endometrioid carcinomas having a more favourable prognosis compared to high grade serous [2] and clear cell carcinomas [3], most likely related to distinct differences in tumour biology [4]. It has been emphasized that each EOC subtype needs to be considered separately in order to identify clinically relevant biomarkers [5]. EOC, and the clear cell subtype in particular, is known to only initially be responsive to chemotherapy treatment [6] and the main prognostic factor remains surgical debulking status [7,8]. This emphasizes that targeted therapies, potentially specific for each subtype, are needed in combination with improved methods for early detection. To identify the biology underlying each clinical subtype and develop new therapeutic targets, gene expression profiling has been used [9,10]. Among others, a new subtype of high grade serous cancer reflecting a mesenchymal cell type, characterized by low expression of MUC1, has been identified. This new subgroup has an undifferentiated phenotype and expresses developmentally associated transcription factors, including SOX11, as well as other high-mobility group members such as HMGA2, TOX and TCF7L1 [11].

SOX11 is a diagnostic and prognostic antigen in B cell lymphomas [12-17] and has recently been demonstrated by us to have tumour suppressor functions [18]. This transcription factor is also a prognostic antigen in EOC, where its presence is associated with improved recurrence-free survival (RFS) [19]. In the present study, we confirm the relationship between SOX11 and survival in EOC, although a larger set of endometrioid cancer needs to be investigated to show independent prognostic relevance. To identify suitable *in vitro *models for functional analyses, EOC cell lines were screened for SOX11 expression and promoter methylation was assessed in both positive and negative cell lines. To verify that methylation is a key event in silencing SOX11, 5-Aza-dC treatment was used to re-express SOX11 in an *in vitro *model of EOC. Furthermore, the tumour suppressor function of SOX11, as previously reported for B cell lymphomas [18], was now extended to EOC and demonstrated through transient over-expression of SOX11.

In summary, we show that SOX11 is a prognostic and functional antigen associated with improved survival in high grade EOC. Furthermore, specific promoter methylation was shown to be a key event in silencing SOX11.

## Methods

### Clinical material and construction of tissue microarrays

The tissue microarray (TMA) was constructed from a consecutive cohort of 154 cases of primary invasive EOC from the prospective, population based cohorts Malmö Diet and Cancer [20] and Malmö Preventive Medicine [21]. The histological re-evaluation of the joint cohort and construction of the TMA has previously been described by Ehlén et al. [22]. The patient cohort is summarised in Table 1. All national and international guidelines including the Helsinki Declaration on ethical principles for medical research involving human subjects, i.e. *Declaration of Helsinki - Ethical Principles for Medical Research Involving Human Subjects *(2000) were applied during the project.

**Table 1:** Clinicopathological characteristics in the total cohort and the SOX11 positive subgroup.

Histology	**N total (%)**^1^	**N SOX11 positive (%)**^2^
Total	154	55
		
*Histological subtype*		
Mucinous	12 (8)^1^	3 (5)
Serous	90 (58)	31 (56)
Endometrioid	35 (23)	17 (31)
Clear cell	9 (6)	0
Brenner	1 (0.6)	0
Unknown	7 (5)	4 (7)
		
*Grade*		
High^3^	107 (70)	40 (73)
Low^4^	47 (30)	15 (27)
		
*Stage*		
I	26 (17)	10 (18)
II	18 (12)	9 (16)
III	75 (49)	26 (47)
IV	22 (14)	5 (9)
Unknown	13 (8)	5 (9)

^1^The percentage is based on the total number of cases.

^2 ^The percentage is based on the total number of SOX11 positive cases.

^3 ^High grade includes cases with low differentiation grade

^4 ^Low grade includes cases with intermediate and high differentiation grade.

### Immunohistochemical analysis of SOX11

TMA sections were pre-treated as previously described [19]. Immunohistochemistry (IHC) was performed, using the primary rabbit anti-human SOX11^C-term ^antibody [13], according to a previous staining protocol [19]. Briefly, signal was detected, using the Dako REAL Detection system and slides were counterstained with Mayers hematoxylin (Sigma-Aldrich, St Louis, MO).

### Image Acquisition, Management and Automated analysis and statistical analysis

The Aperio ScanScope XT Slide Scanner (Aperio Technologies, Vista, CA) system was used to capture whole slide digital images with a 20X objective and nuclear SOX11 expression was automatically quantified. A tumor specific nuclear algorithm (*IHC -MARK*) was developed in house to quantify SOX11 protein expression. *IHC-MARK *was designed to identify tumor cells on the basis on nuclear morphology and disregard non-tumor cells such as normal epithelial or stromal cells, or invading leukocytes as previously described [23]. The algorithm calculated the percentage of positive tumor cells, as well as relative staining intensity ranging from 0 to 255. Patients were divided into subgroups having either higher or lower than 10% nuclear SOX11 expression. Statistical analysis was performed, as previously described [19]. Briefly, the χ^2 ^test was used for comparison of SOX11 expression and clinicopathological data, and Kaplan-Meier analysis and the log rank test were applied to evaluate and illustrate ovarian cancer specific survival (OCSS) and overall survival (OS) in strata, according to SOX11 expression. Cox regression proportional hazards models were used to estimate the relationship between SOX11 expression in high grade tumours and stage. All calculations were performed, using SPSS version 11.0 (SPSS Inc, Chicago, IL).

### Cultivation of EOC cell lines

In total, five different EOC cell lines (Table 2) were used in the study: TOV-112D, derived from malignant solid epithelial ovarian tumour specimens [24], NIH:OVCAR-3, originating from the malignant ascites of a patient with progressive adenocarcinoma [25], ES-2 [26], A2780 and A2780-CP7 [27]. The cell line TOV-112D was cultured in DMEM High Glucose media (Hyclone, South Logan, Utah, USA) supplemented with 15% fetal bovine serum (FBS) and 1% L-glutamin (both Invitrogen, Carlsbad, CA, USA). OVCAR-3 was maintained in RPMI-1640 media (HyClone) supplemented with 20% FBS, 1% L-glutamin and 0,01 mg/ml insulin (Sigma-Aldrich). A2780 and A2780-CP7 were maintained in RPMI-1640 media supplemented with 10% FBS and 1% 200 mM L-glutamine. ES-2 was cultured in McCoy's 5A media (HyClone) supplemented with 10% FBS.

**Table 2:** EOC Cell line characteristics

Cell line	Disease
TOV-112D^1^	Primary malignant adenocarcinoma
OVCAR-3^3^	Progressive adenocarcinoma
ES-2^1^	Poorly differentiated ovarian clear cell carcinoma
A2780^2^	Undifferentiated CA
A2780-CP7^2^	Undifferentiated CA

1 - Kindly provided by Gabriella Honeth, Department of oncology, Lund University

2 - Kindly provided by who Prof R Brown, Imperial College, London

3 - American Type Culture Collection (ATCC)

### RNA isolation and quantitative real time PCR

The relative quantity (RQ) of *SOX11 *mRNA in various cell lines was identified using Real Time-quantitative PCR (qRT-PCR). The cells were lysed and cDNA synthesis performed using the Fast SYBR Green Cells-to-CT kit and the samples were run on a Fast 7500 qRT-PCR system (Applied Biosystems, Foster City, CA, USA), as previously described [18]. Briefly, 30 000-50 000 cells were washed in PBS, lysed and treated with DNase. Lysates were reversed-transcribed and cDNA amplified in three technical replicates with the following primer specific either for *SOX11 *or the house-keeping gene GAPDH (the concentration was 250 nM (MWG, High-Point, NC, USA)); GAPDH: 5'-TGGTATCGTGGAAGGACTC-3' and 5'-AGTAGAGGCAGGGATGATG-3', *SOX11*: 5'-GGTGGATAAGGATTTGGATTCG-3' and 5'-GCTCCGGCGTGCAGTAGT-3'. The RQ is calculated as 2^-(ΔΔCT(SOX11-GAPDH))^. For analysis of absolute levels of SOX11, a control sample was run, containing lysate but no reverse transcriptase (RT), enabling estimation of amplification above genomic level, as previously described [18]. Generally, all samples with a ΔC_T (SOX11+RT, SOX11-RT) _< |2|, here after referred to as ΔC_T,SOX11, _were considered negative. All the error bars related to qRT-PCR data have been calculated using the standard error (SE) value with a 95% confidence interval.

### Protein purification and quantification

Protein was extracted and quantified as previously described [18]. Briefly, 0.2 to 8 million cells were harvested, washed and placed in 200 μl lysis-buffer (1% Ipegal/Protease Inhibitor cocktail (Roche, Basel, Switzerland) in PBS).

### Western Blot analysis of *SOX11*-knockdown and differential expression

Protein lysates, 43 μg for wild-type expression, lysates of 0.2 million cells for demethylation and 30 μg for overexpression, from five ovarian cancer cell lines were run on NuPAGE 10% Bis-Tris gels (Invitrogen, Carlsbad, CA, USA) under reducing conditions for ~45 min at 130 V. Separated proteins were blotted on PVDF iBlot Transfer stacks in the iBlot gel transfer device (both Invitrogen) and blocked for 1 h. SOX11 protein expression was verified using an antibody targeting SOX11 (Atlas Antibodies, Stockholm Sweden) and a HRP-labelled swine anti-rabbit antibody (DAKO, Glostrup, Denmark) as secondary antibody. Blots were developed, using the SuperSignal West Femto Maximum Sensitivity Substrate (Pierce, Thermo Scientific, Waltham, MA, USA), according to the protocol of the manufacturer, detected in a CCD-camera and analysed in the Quantity One software (both Bio-Rad, Hercules, CA, USA).

### Analysis of promoter methylation status

The methylation status of the SOX11 promoter was determined by sodium bisulfite sequencing and MSP. Briefly, DNA was extracted from the cell lines with QIAamp DNA Mini Kit (QIAgen, Hilden, Germany). 0.5-1 μg of DNA was bisulfite converted with QIAgen Epitect Bisulfite Kit (QIAgen) and 50-100 ng of converted DNA was used as template in the PCR. Epitect Control DNA (QIAgen) was used as controls in all PCR reactions to ensure specific PCR amplification using Platinum Taq DNA polymerase (Invitrogen). For bisulfite sequencing, primers that amplified a 213 bp region containing 28 CpG's adjacent to the 5' end of the SOX11 transcription start site were used as previously described [18]. PCR products were directly sequenced by Eurofins MWG Operon (Ebersberg, Germany). Clonal analysis of individual alleles was made by subcloning the PCR fragments into pCR.21-TOPO vector (Invitrogen) and transformed into chemically competent E.coli TOP10 (Invitrogen). MSP was performed with primers specific for methylated and unmethylated bisulfite converted DNA in the same region as the bisufite sequencing primers. The primers were *5'-TAG TCG CGT TTT TAA ATA TTA TCG A-3' *(M-fwd) *5'-CCT AAC CGA CGA AAA ATA ACG-3' *(M-rev) and *5'-TAG TTG TGT TTT TAA ATA TTA TTG A-3' *(U-fwd) *5'-ACC CTA ACC AAC AAA AAA TAA CAC T-3' *(U-rev). PCR products were analyzed on a 2% agarose gel.

### Demethylation experiments using 5'-Aza-2'deoxycytidine

To perform demethylation experiments, the ovarian cancer cell line ES-2 that is methylated in the SOX11 promoter was used. ES-2 was seeded and grown o/n until 60% confluency. 5-Aza-dC (Sigma Aldrich, St. Louis, MO, USA) was applied (2μM or 10μM) to the wells together with fresh media every 24^th ^hours. Equivalent amount of media was added to mock-treated cells. After 96 hours the cells were harvested by trypsination. DNA and protein was extracted as above. Quantification of DNA and protein was performed using the volume measurement (Intensity*Area) and global background subtraction in Quantity One software (Bio-Rad). For DNA quantification, the total volume of pairwise DNA samples (treated and untreated control samples) was set to 100%. For protein quantification, the relative volume of untreated control sample was set to 1.

### Overexpression of SOX11 in EOC cell lines

Transfection of the cells to overexpress SOX11 was performed following the Lipofectamine™ 2000 protocol (Invitrogen). Briefly, one to two days prior to transfection 50 000 - 100 000 cells/cm^2 ^from each cell line were seeded into culture vessels. Transfection was performed with the SOX11-GFP plasmid, OmicsLink™ Expression Clone, EX-M0425-M46 (GeneCopoeia™), in one culture vessel and with the GFP plasmid; OmicsLink™ Expression Clone, EX-EGFP-M02 (GeneCopoeia™); in another with a DNA (μg):Lipofectamine™ 2000 (μl) ratio of 1:2.5. 24 hours after transfection the cells were harvested and samples were taken for mRNA, protein and proliferation assays.

### Proliferation assay using [methyl-3H]-thymidine incorporation and assessment of confluency

The proliferation assays were performed using [methyl-3H]-thymidine incorporation. 15000 cells were seeded in triplicates onto 96-well plates, left for a couple of hours for the cells to attach, then [3H]-thymidine (1 mCi/ml, 5 Ci/mmol, Amersham, GE Healthcare) was added and the cells were incubated for 8-16 hours. Cells were then frozen and finally thawed and harvested in a Tomtec Harvester 96 (Tomtec, Hamden, CT, USA) and analysed with a 1450 MICROBETA liquid scintillation counter (Wallac (now PerkinElmer^®^, Waltham, MA, USA)). To assess confluency, treated and control cell lines were photographed 48 h after overexpression of SOX11 using a Fluovert FS inverted microscope (Leitz, MI, United States) with 32× magnification and a Nikon Coolpix 995 digital camera (Nikon, Tokyo, Japan).

## Results

### SOX11 expression and correlation to survival in high grade and endometrioid carcinomas

**Figure 1:**
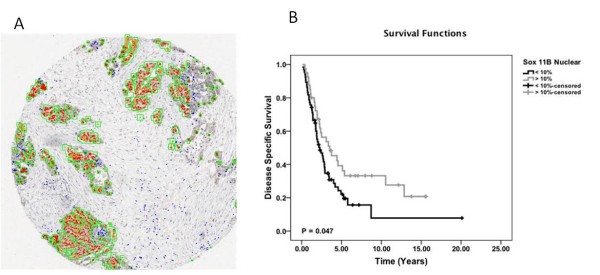
**IHC and survival analyses of SOX11**. (A) Representative nuclear SOX11 staining identified using an automatic tumor specific algorithm showing nuclear expression in red. (B) Kaplan-Meier analysis and the log rank test identified SOX11 as significant associated (p = 0.047) with cancer specific survival in high grade tumours, including tumours with low differentiation grade. The patients were classified into two groups based on SOX11 expression.

SOX11 was recently discovered to be a diagnostic [12-14,16], prognostic [15,17] and functionally associated antigen [18] in B cell lymphomas, but also of prognostic value in EOC [19]. To further investigate the importance of SOX11 expression in EOC, we used a large TMA comprising different histological variants, such as mucinous, serous, endometrioid and clear cell [22,28]. A tumor specific nuclear algorithm (IHC-MARK) was developed and used to evaluate the nuclear SOX11 protein expression. A representative IHC staining showing nuclear expression of SOX11 is shown in Figure 1a. Kaplan Meier estimates demonstrated an association between SOX11 nuclear expression and an improved cancer-specific survival in high grade EOC (p = 0.047) (Figure 1b). Multivariate analysis demonstrated a co-dependence with stage. Analysis of SOX11 expression in relation to histological subtypes revealed a non-significant trend towards an improved overall survival (OS) within the endometrioid subtype (p = 0.066) (data not shown)). In contrast to previous data where SOX11 mRNA was shown to specifically be expressed in high grade serous cancer [11], our analyses showed no correlation between SOX11 status and distribution of high and low grade tumours in individual histological subtypes (data not shown).

### SOX11 promoter methylation status correlates to mRNA and protein expression

**Figure 2:**
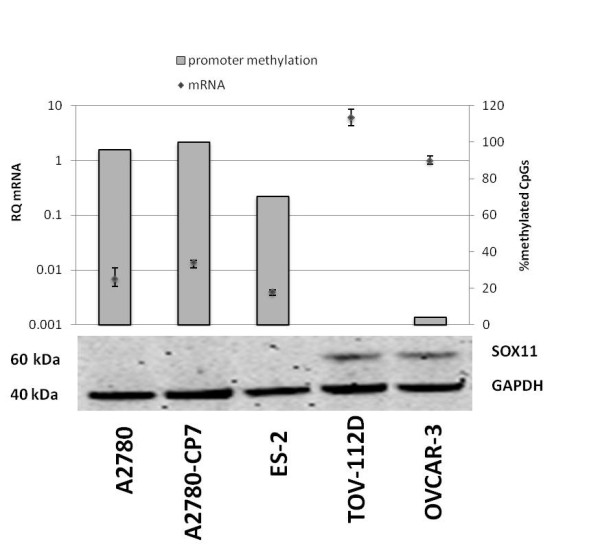
**Promoter methylation of SOX11 correlates to mRNA and protein expression in EOC cell lines**. The relative mRNA levels (RQ) as compared to OVCAR-3 are shown as diamonds. The standard deviation for the RQ values has been calculated with a 95% confidence interval (CI). Below, corresponding protein levels are shown. The degree of SOX11 promoter methylation, described as percentage of methylated CpGs of 28 possible CpG methylation sites, is shown as bars. The three cell lines A2780, A2780-CP7 and ES-2 all lack mRNA and protein expression of SOX11, correlating to a high percentage of methylated CpG sites.

In an attempt to understand the functional implications of SOX11 expression in EOC, we analysed the expression of both SOX11 mRNA and protein in five different EOC cell lines. TOV-112D and OVCAR-3 were shown to have significant levels of SOX11 expression (Figure 2) as compared to the other cell lines. Of interest, TOV-112D is of endometrioid origin and the expression of SOX11 in this cell line is consistent with the presence of primary SOX11-positive endometrioid tumours (Table 1). The origin of OVCAR-3, which also expressed SOX11, is unfortunately not known. To determine the methylation status of the SOX11 promoter in all five EOC cell lines sodium bisulfite sequencing was used. 28 CpG's in the SOX11 promoter were analysed (Table 3) and the percentage of methylated CpG's calculated (Figure 2). The three cell lines A2780, A2780-CP7 and ES-2 were highly methylated which correlated with a complete lack of SOX11 expression at both mRNA and protein level. The two cell lines TOV-112D and OVCAR-3, on the other hand, were demethylated in the SOX11 promoter, which correlated to expression of SOX11 at both the mRNA and protein level.

**Table 3:** Methylation status of individual CpGs in EOC cell lines

Cog position	A2780	A2780-CP7	ES-2	TOV-112D	OVCAR-3
CpG1	NA ^1^	NA	UM	UM	NA
CpG2	NA	NA	NA	UM	NA
CpG3	**M **^2^	NA	UM	UM	NA
CpG4	UM ^3^	NA	UM	UM	NA
CpG5	**M**	**M**	**M**	UM	**M**
CpG6	**M**	**M**	**M**	UM	UM
CpG7	**M**	**M**	UM	UM	UM
CpG8	**M**	**M**	**M**	UM	UM
CpG9	**M**	**M**	UM	UM	UM
CpG10	**M**	**M**	**M**	UM	UM
CpG11	**M**	**M**	UM	UM	UM
CpG12	**M**	**M**	UM	UM	UM
CpG13	**M**	**M**	**M**	UM	UM
CpG14	**M**	**M**	**M**	UM	UM
CpG15	**M**	**M**	UM	UM	UM
CpG16	**M**	**M**	**M**	UM	UM
CpG17	**M**	**M**	**M**	UM	UM
CpG18	**M**	NA	**M**	UM	UM
CpG19	NA	**M**	**M**	UM	UM
CpG20	**M**	**M**	**M**	UM	UM
CpG21	**M**	**M**	**M**	UM	UM
CpG22	**M**	**M**	**M**	UM	UM
CpG23	**M**	**M**	**M**	UM	UM
CpG24	**M**	**M**	**M**	UM	UM
CpG25	**M**	**M**	**M**	UM	UM
CpG26	**M**	**M**	**M**	UM	UM
CpG27	**M**	**M**	**M**	UM	UM
CpG28	**M**	**M**	**M**	UM	UM

^1^NA - sequence data not available

^2^**M **- methylated CpG positions are shown in bold

^3^UN - unmethylated CpG positions

### Demethylation of ovarian cancer cell line ES-2 induced SOX11 mRNA and protein expression

**Figure 3:**
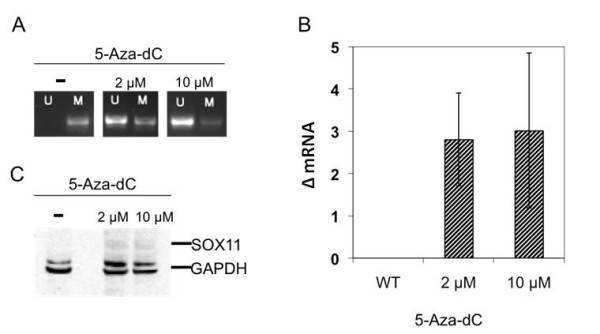
**Induced expression of SOX11 in ES-2**. (A) Treatment of the ovarian cancer cell line ES-2 with 5-Aza-dC for 96 h led to demethylation of the SOX11 promoter. (B) SOX11 mRNA can be detected above background level by qRT-PCR. (C) SOX11 protein expression as determined by Western Blot analysis. The data shown here is a representative example of three independent experiments.

The SOX11 negative epithelial ovarian cancer cell line ES-2 is methylated in the SOX11 promoter region. In order to demonstrate that the methylation is responsible for silencing of the *SOX11 *gene, this cell line was treated with the demethylating agent 5-Aza-dC. Treatment of ES-2 with 2 and 10 μM 5-Aza-dC for 96 hours resulted in demethylation of the *SOX11 *promoter (Figure 3A). Quantification showed an increase from 8% (untreated control) to 57% (2 μM 5-Aza-dC) and 82% (2 μM 5-Aza-dC) unmethylated DNA. This was accompanied by an immediate upregulation of SOX11 mRNA (Figure 3B). Also analysis on the protein level confirmed expression of SOX11 and quantification showed a 4.6 and 3.5 fold increase in protein level comparing treated (2 and 10 μM 5-Aza-dC, respectively) with untreated samples (Figure 3C). Analysis of the absolute levels of SOX11 in wt SOX11-positive and negative EOC cell lines showed that TOV-112D and OVCAR-3 had a ΔC_T,SOX11 _of 5.4 and 7.0 respectively, while ΔC_T,SOX11 _were <1.0 for the negative cell lines. Thus, it is clear that the demethylation resulted in significant amounts of SOX11, but at a level lower than the wt positive cell lines. Thus, SOX11-negative ovarian cancer cell lines are specifically silenced by DNA methylation that at least partly can be reversed by demethylating drugs.

### Overexpression of SOX11 in EOC cell lines

**Figure 4:**
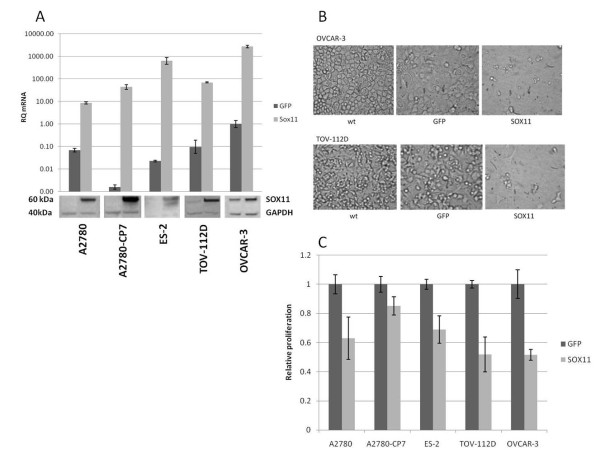
**Overexpression of SOX11 induces growth arrest in EOC cell lines**. (A) The relative mRNA levels of SOX11 24 h after overexpression in different cell lines, as compared to control cells (OVCAR-3 transfected with GFP containing plasmid). The standard deviation for the RQ values has been calculated with a 95% CI. Below, corresponding protein levels for control (GFP) and SOX11-transduced cell lines after 24 h are shown. (B) The confluency of the transfected cell lines OVCAR-3 and TOV-112D decreased markedly after overexpression of SOX11 compared to GFP control and untreated wt cells, consistent with a growth inhibitory role of SOX11. (C) The relative proliferation with the control (GFP) samples as reference for each cell line is shown. A decrease in proliferation in all cell lines was seen 24 h after overexpressing SOX11.

To further evaluate the functional role of SOX11 the gene was overexpressed in five different EOC cell lines, by transfecting them with a SOX11-GFP plasmid (OmicsLink™ Expression Clone). Transfection efficiency was 40-90%, measured as GFP positive control cells in flow cytometry (data not shown). Both mRNA and protein extracts were analysed by qRT-PCR and western blot to confirm an overexpression of SOX11. Furthermore, the proliferation of the cells was assessed by thymidine incorporation. The level of overexpression at mRNA level varied between a 100-fold (A2780) to a 50000-fold (A2780-CP7). For most cell lines the increase in mRNA was also translated into an overexpression of the SOX11 protein. However, the 1000-fold increase of mRNA in OVCAR-3 only resulted in a limited increase of SOX11 protein (Figure 4A). Using light microscopy to investigate the confluency of the cell lines, it was evident that the induction of SOX11 overexpression resulted in a significant decrease in cell number (Figure 4B), as compared to the control. Furthermore, the overexpression of SOX11 resulted in a decrease in proliferation in all cell lines (Figure 4C). There was not a linear correlation between level of overexpression and reduction in cell proliferation. Among others, OVCAR-3, with a modest increase of SOX11 protein responded with >50% reduced proliferation, while A2780-CP7, with the strongest overexpression showed a lower decrease in growth rate. The difference in response to SOX11 overexpression comparing different cell lines may be related to the need of co-factors, as the strongest effect was seen in cell lines which already expressed SOX11, as also has been reported for B cell lymphomas [18].

## Discussion

SOX11 has previously been identified as a prognostic antigen in EOC, where SOX11 expression was associated with a prolonged recurrence free survival [19]. In this study, we demonstrated that SOX11 was associated with an improved survival among patients with high-grade carcinomas and potentially also with endometrioid carcinomas, although the latter needs to be statistically confirmed in a larger cohort of patients. It is well known that EOC constitutes a diverse set of malignancies, each with a separate histology, gene expression pattern [29] and outcome tightly linked to the underlying biology [4]. It has been shown that low malignant potential tumours often progress to low grade serous carcinomas but that high grade tumours rapidly develop from surface epithelium without evidence of previous lesion [30]. This is consistent with a stepwise mutation process (low-grade pathway) or a greater genetic instability (high-grade pathway), as previously reviewed [31]. It is of major interest that SOX11 can be used to further subdivide high grade tumours, which also indicates a functional role for SOX11 as a tumour suppressor. It is not clear whether SOX11 defines endometrioid carcinoma of a specific origin, since it has been suggested that they, like clear cell ovarian cancer, may be derived from endometriotic deposits in contrast to the surface epithelial layer of the ovary or distal fallopian tube [32]. Of importance, SOX11 is expressed in early progenitor human multipotent stromal cells but expression decreases with expansion of the cells [33]. Further studies are needed to determine if SOX11 also is associated with differentiation pattern in endometrioid carcinomas, although they are known to mostly be well differentiated [34], in contrast to our cohort. Preliminary, no association with differentiation grade of endometrioid carcinomas and SOX11 expression was seen, although the number of patients was limited. More apparent, SOX11 expression was not restricted to a specific histological subtype, however most SOX11 positive cases were found in the serous and endometrioid subtypes. Correlation to the *in vitro *models could not be performed as the majority of these are poorly characterized. Some of the cell lines we used have previously been xenografted into nude mice to analyse the resulting pathologies [27], but no firm conclusion can be drawn between any correlation of SOX11 and their potential histological subtype.

As previously shown for B cell lymphoma cell lines [18], the protein and mRNA expression of SOX11 in ovarian cancer cell lines correlated with the methylation status of the SOX11 promoter region. Given the tumour suppressor function of SOX11 [18] and the association with cell cycle status [35], which has been demonstrated previously by us in lymphomas, it is not surprising that the promoter region of SOX11 in some EOC cell lines becomes methylated in order to silence gene expression. In ovarian cancer cell lines, knock-down of DNMT1 and DNMT3b, resulting in loss of CpG hypermethylation, has a negative effect on growth [36]. The specific methylation of tumour suppressors is a known event in many different cancers and is commonly studied by forced demethylation through agents such as 5-Aza-dC, that inhibit the DNA methyl transferases (DNMT's) [37,38] and subsequent analysis of expression of target genes. Here, we were able to demethylate the SOX11 promoter in the ovarian cancer cell line ES-2, resulting in a successful expression of SOX11 mRNA and protein. However, the level of SOX11 expression upon demethylation was significantly lower compared to SOX11 wt positive control cell lines such as TOV-112D and OVCAR-3. Probably, there are additional epigenetic factors besides an unmethylated promoter that are needed to reach the endogenous levels of wt SOX11 expression. Others have recently shown that SAHA treatment used in combination with 5-Aza-dC increase the expression of SOX11 in a negative B cell lymphoma cell line [39]. In ovarian cancer, platinum-resistance is a common problem and it has been proposed that this might in part be explained by hypermethylation and other epigenetic events that repress tumour suppressor genes in general [40]. Thus, the successful use of DNMT and HDAC (Histone deacetylases) inhibitors for chemotherapy re-sensitization in ovarian cancer cell lines and animal models imply that epigenetic therapies could be a future method for clinical chemo re-sensitization [40]. This is also supported by our data where forced SOX11 expression leads to a reduced proliferation.

Of interest, the clinical outcome of patients afflicted by EOC has been associated to differential expression of cell cycle regulatory proteins including Cyclin D1, Cyclin E, p16-INK and E2F transcription factors [41]. We have previously shown that some of these proteins, including p16INK, Rb and E2F transcription factors [18] change their expression, as a result of overexpression of SOX11 in lymphoma cell lines and these genes may thus be responsible for the improved survival associated with SOX11-positive tumours also in EOC.

## Conclusions

In the present study, SOX11 was demonstrated to be of prognostic value for high grade EOC, which could have a clear clinical value. The possibility to re-express SOX11 indicates a potential use of epigenetic drugs to affect cell growth through common cell regulatory pathways, controlled by SOX11, and other tumour suppressors that are silenced in EOC. Furthermore, functional investigations *in vitro *confirmed a growth regulatory role for SOX11 in EOC.

## Competing interests

A patent has been filed on the use of SOX11 as a diagnostic and prognostic antigen in EOC.

## Authors' contributions

SS performed experimental work, including overexpression studies, analysis of the cell lines and took active part in writing the manuscript. EG performed experimental work in relation to promoter methylation analysis and took active part in writing the manuscript. DJB was responsible for the design of the automated SOX11 classification, interpretation of the data and involved in the completion of the manuscript. WMG and ER were involved in the design of the algorithm for automated IHC classification. FR performed experimental work in relation to overexpression of SOX11. KJ provided the tumour material and clinical data and was involved in the completion of the manuscript. CB was involved in the design of the study and the completion of the manuscript. SE was responsible for the design of the study, interpretation of data and writing of the manuscript. All authors read and approved the final manuscript.

## Pre-publication history

The pre-publication history for this paper can be accessed here:

http://www.biomedcentral.com/1471-2407/11/405/prepub
